# Endoscopic Ultrasonography Assessment for Ampullary and Bile Duct Malignancy

**DOI:** 10.1155/DTE.3.35

**Published:** 1996

**Authors:** Q. L. Zhang, W. D. Nian, L. P. Zhang, J. Y. Liang

**Affiliations:** Department of Surgery The First Teaching Hospital Beijing Medical University Beijing (100034) China

## Abstract

From 1989 through 1992, endoscopic ultrasonography (EUS) was undertaken preoperatively to evaluate the extent of primary tumor, involvement of regional lymph nodes, and distant metastases in 22 patients with ampullary carcinoma and 18 patients with bile duct carcinoma. The results were compared with histopathological findings according to the TNM staging system. The accurate rate in assessing the extent of cancer invasion was 82% for ampullary carcinoma, 66% for common hepatic duct carcinoma, and 78% for common bile duct carcinoma. The accuracy of EUS in predicting regional lymph node metastasis was 59% for ampullary carcinoma, 56% for common hepatic duct carcinoma, and 67% for common bile duct carcinoma. Invasion of the portal vein was correctly predicted by EUS in 2 of 3 patients. None of the 3 patients with liver metastasis was detected by EUS. Therefore, endoscopic ultrasonography is an effective method in the evaluation of the extent of cancer invasion of ampullary and bile duct carcinoma as well as the involvement of regional lymph nodes preoperatively. However, due to its limited penetration depth, EUS is inadequate in the assessment of liver metastasis.


*Diagnostic and Therapeutic Endoscopy*, Vol. 3, pp. 35–40
Reprints available directly from the publisher
Photocopying permitted by license only

© 1996 OPA (Overseas Publishers Association)
Amsterdam B.V. Published in The Netherlands
by Harwood Academic Publishers GmbH
Printed in Singapore

### Endoscopic Ultrasonography Assessment for Ampullary and Bile Duct Malignancy

Q. L. ZHANG*, W. D. NIAN, L. P. ZHANG and J. Y. LIANG

*Department of Surgery, The First Teaching Hospital, Beijing Medical University, Beijing (100034), P. R. China*

*(Received 21 November 1994; In final form 22 March 1996)*

**From 1989 through 1992, endoscopic ultrasonography (EUS) was undertaken preoperatively to evaluate the extent of primary tumor, involvement of regional lymph nodes, and distant metastases in 22 patients with ampullary carcinoma and 18 patients with bile duct carcinoma. The results were compared with histopathological findings according to the TNM staging system. The accurate rate in assessing the extent of cancer invasion was 82% for ampullary carcinoma, 66% for common hepatic duct carcinoma, and 78% for common bile duct carcinoma. The accuracy of EUS in predicting regional lymph node metastasis was 59% for ampullary carcinoma, 56% for common hepatic duct carcinoma, and 67% for common bile duct carcinoma. Invasion of the portal vein was correctly predicted by EUS in 2 of 3 patients. None of the 3 patients with liver metastasis was detected by EUS. Therefore, endoscopic ultrasonography is an effective method in the evaluation of the extent of cancer invasion of ampullary and bile duct carcinoma as well as the involvement of regional lymph nodes preoperatively. However, due to its limited penetration depth, EUS is inadequate in the assessment of liver metastasis.**

*Keywords:* Endoscopic ultrasonography, carcinoma, papilla of Vater, bile duct

### INTRODUCTION

Surgery is still the main method for curing ampullary and bile duct carcinoma at the present time. Although the 5-year survival rate after a Whipple operation for ampullary and distal end bile duct carcinoma is about 50%, there is also a 5 to 10% mortality after this operation. A correct preoperative evaluation of the extent of the primary tumor, involvement of regional lymph nodes, and distant metastases will contribute to choosing an appropriate therapeutic method and determining the prognosis.

Since 1989, we have used endoscopic ultrasonography (EUS) assessment for ampullary and bile duct carcinoma preoperatively, and compared the results with the surgical explorations and pathological findings for resected specimens for evaluating the accuracy of EUS according to the TNM staging system [1].

*Corresponding author. Tel.: 86-1-6031122-2437. Fax: 86-1-6037106.

### PATIENTS AND METHODS

### Subjects

From 1989 through the end of 1992, 22 patients with ampullary carcinoma (15 men and 7 women aged 18 to 72 years, mean 50.4 years) and 18 patients with bile duct carcinoma (10 men and 8 women aged 36 to 72 years, mean 61.2 years) were examined by EUS preoperatively. All 40 patients underwent surgical exploration, and all the lesions were resected. The results of EUS assessment were compared with the histological findings of the resected specimens. In addition to the 40 patients with resectable tumor, there were other 6 patients with nonresectable tumor. Among these patients, invasion of portal vein was found in 3 patients, with 2 of 3 patients correctly predicted by EUS. Liver metastasis was found in 3 patients, and none of these were detected by EUS.

### Instrument

All studies were performed with an echoendoscope (Olympus GF-UM2 and GF-UM3). A water-filled balloon or a combination with the water-filling method was used for getting a better acoustic coupling between the transducer and the mucosa.

### Manipulative Method

The echoendoscope was inserted into the second portion of the duodenum closing to the ampulla of Vater. After identification of the common bile duct and the pancreatic head using the portal vein as a landmark, the echoendoscope was gradually withdrawn. Careful scanning and assessment were done for the extent of tumor, enlarged regional lymph nodes, and distant metastasis.

### Interpretation of Endosonography

Ampullary cancer was diagnosed when a hypoechoic mass was imaged in the region of ampulla of Vater with destruction of the normal structure of ampulla, with or without infiltration into the pancreas or other surrounding structures (Fig. 1).

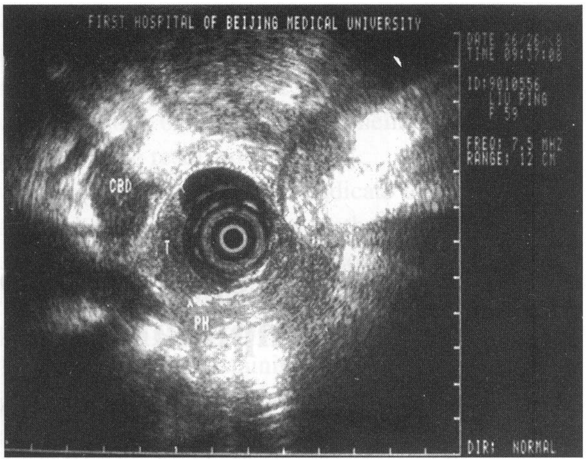

FIGURE 1 EUS image shows a hypoechoic tumor (T) destroying the whole layer structures of the papilla of Vater and invading the pancreatic head (PH) with dilatation of the common bile duct (CBD).

Bile duct carcinoma was imaged as a hypoechoic or hyperechoic inhomogeneous lesion with or without invading the surrounding structures (Fig. 2).

Lymph nodes with a hypoechoic pattern and clearly delineated margin were generally considered as malignant. Lymph nodes with a hyperechoic pattern and an uncleared margin were considered as benign.

### RESULTS

Among the 40 patients in whom malignant lesions were resected completely and the results of EUS assessment were compared with the histological findings for resected specimens, the accuracy rate for assessing the extent of cancer invasion was 82% for ampullary carcinoma (Table I), 66% for common hepatic duct carcinoma (Table II), and 78% for common bile duct carcinoma (Table III).

There were other 6 patients with nonresectable tumor due to invasion of portal vein or liver metastasis. Invasion of the portal vein was correctly predicted by EUS in 2 of 3 patients. None of the 3 patients with liver metastasis was detected by EUS.

The accuracy of EUS in predicting the regional lymph node metastasis was 59% for ampullary carci-

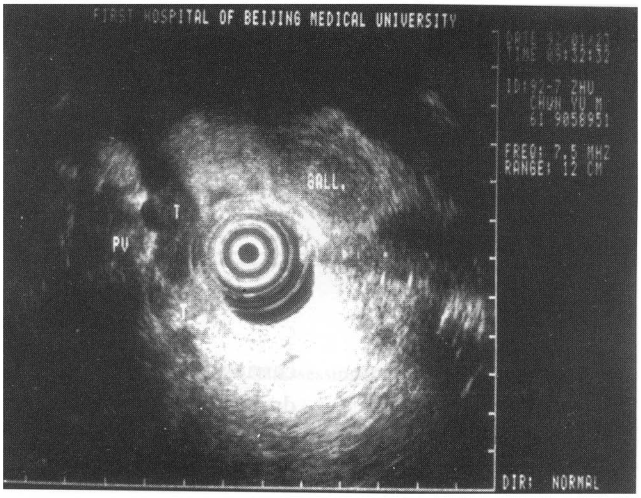

FIGURE 2 EUS image shows a hypoechoic tumor (lower T) at the end of dilated common bile duct with strongly suspected infiltration of portal vein (PV) as well as the middle portion of common bile duct (upper T).

TABLE I Results of Pathologic Staging and EUS in Assessing the Extent of Ampullary Carcinoma*

| Pathologic | | EUS | | | |
|---|---|---|---|---|---|
| Staging | *n* | Correct Staging | Overstaging | Understaging | Accuracy (%) |
| pT1 | 2 | 2 | | | 100 |
| pT2 | 16 | 14 | | 2 | 88 |
| pT3 | 4 | 2 | | 2 | 50 |
| Total | 22 | 18 | | 4 | 82 |

*According to TNM classification.

TABLE II Results of Pathologic Staging and EUS in Assessing the Extent of Common Hepatic Duct Carcinoma*

| Pathologic | | EUS | | | |
|---|---|---|---|---|---|
| Staging | *n* | Correct Staging | Overstaging | Understaging | Accuracy (%) |
| pT1 | 0 | | | | |
| pT2 | 4 | 3 | | 1 | 75 |
| pT3 | 5 | 3 | | 2 | 60 |
| Total | 9 | 6 | | 3 | 66 |

*According to TNM classification.

TABLE III Results of Pathologic Staging and EUS in Assessing the Extent of Common Bile Duct Carcinoma*

| Pathologic | | EUS | | | |
|---|---|---|---|---|---|
| Staging | n | Correct Staging | Overstaging | Understaging | Accuracy (%) |
| pT1 | 0 | | | | |
| pT2 | 5 | 4 | 1 | | 80 |
| pT3 | 4 | 3 | | 1 | 75 |
| Total | 9 | 7 | 1 | 1 | 78 |

*According to TNM classification.

noma (Table IV), 56% for common hepatic duct carcinoma (Table V), and 67% for common bile duct carcinoma (Table VI).

### DISCUSSION

The Whipple operation is the standard method for curing ampullary and distal end bile duct carcinoma. The 5-year survival rate after this operation for ampullary carcinoma is about 50% [2], and it also carries a 5% mortality even in expert hands [3,4]. So it is very important to evaluate the extent of primary tumor as well as to rule out major vascular involvement and distant metastases before surgery, that is, to select a patient with a resectable tumor for this operation. For elderly patients, if the extent of ampullary carcinoma is still within the duodenal wall, sometimes a local resection is much better and safer. If invasion of major vascular structures or distant metastases are detected before surgery, which means the carcinoma is unresectable, then an alternative nonsurgical palliative procedure, such as endoscopic biliary stenting, will be available for them, because it carries a very low morbidity and mortality and can drain the bile juice well.

EUS has been under development for more than a decade. It now plays a well-established role in the staging for gastrointestinal tract malignancies preoperatively. It has an accuracy of 83 to 88% and 82 to 86% in determining the extent of ampullary carcinoma and bile duct carcinoma, respectively [5–8]. The accuracy rate in our group was 82% in assessing the extent of ampullary carcinoma, and 66% in common hepatic duct carcinoma, and 78% in common bile duct carcinoma. Understaging of ampullary and bile duct carcinoma might be due to microscopic infiltration or incorrect assessment of the infiltration. Overstaging of bile duct carcinoma by EUS might be due to compression of the blood vessels simulating infiltration by tumor mass.

The overall accuracy of EUS in predicting lymph node metastases was 54 to 92% and 53 to 65% in ampullary carcinoma and bile duct carcinoma, respectively [5–8]. The accuracy in our group was 59% in ampullary carcinoma, 56% in common hepatic duct carcinoma, and 67% in common bile duct carcinoma. The accuracy of EUS in assessing lymph node metas-

TABLE IV Results of Pathologic Staging and EUS in Assessing Regional Lymph Node Metastasis of Ampullary Carcinoma

| Pathologic | | EUS | | | |
|---|---|---|---|---|---|
| Staging | n | Correct Diagnosis | False-Positive | False-Negative | Accuracy (%) |
| pN0 | 12 | 8 | 4 | | 67 |
| pN1 | 10 | 5 | | 5 | 50 |
| Total | 22 | 13 | 4 | 5 | 59 |

*According to TNM classification.

TABLE V Results of Pathologic Staging and EUS in Assessing Regional Lymph Node Metastasis of Common Hepatic Duct Carcinoma

| Pathologic | | EUS | | | |
|---|---|---|---|---|---|
| Staging | n | Correct Diagnosis | False-Positive | False-Negative | Accuracy (%) |
| pN0 | 4 | 3 | 1 | | 75 |
| pN1 | 5 | 2 | | 3 | 40 |
| Total | 9 | 5 | 1 | 3 | 56 |

*According to TNM classification.

TABLE VI Results of Pathologic Staging and EUS in Assessing Regional Lymph Node Metastasis of Common Bile Duct Carcinoma

| Pathologic | | EUS | | | |
|---|---|---|---|---|---|
| Staging | n | Correct Diagnosis | False-Positive | False-Negative | Accuracy (%) |
| pN0 | 3 | 3 | | | 100 |
| pN1 | 6 | 3 | | 3 | 50 |
| Total | 9 | 6 | | 3 | 67 |

*According to TNM classification.

tases for both ampullary carcinoma and bile duct carcinoma is not very high. In general, lymph nodes with a hypoechoic pattern and clearly delineated margin were considered as malignant; lymph nodes with a hyperechoic pattern and indistinct margin were considered as benign. However, sometimes it is difficult to distinguish a malignant lymph node metastasis from a nonmetastatic lymph node abnormality. Criteria for defining metastatic as well as nonmetastatic lymph nodes should be improved for increasing the accuracy of EUS.

Invasion of the portal vein in ampullary and bile duct carcinoma was correctly detected by EUS in 2 of 3 patients. But none of the 3 patients with liver metastases was detected by EUS due to its limited penetration depth.

### CONCLUSION

EUS is accurate for assessing the extent of primary tumors of ampullary and bile duct carcinoma. It is satisfactory in predicting regional enlarged lymph nodes, but sometimes it is difficult to distinguish their nature. EUS is inadequate for evaluating distant metastases of ampullary and bile duct carcinoma, especially in the liver. It is safer than endoscopic retrograde cholangiopancreatography for a patient with malignant obstructive jaundice.

### *Acknowledgments*

This article was presented at the 3rd International Symposium of Therapeutic Endoscopy on Bilio-Pancreatic Diseases on September 23, 1993 in Kobe, Japan.

### *References*

[1] American Joint Committee on Cancer, Beahrs, O. H., Henson, D. E., *et al.* (1988). Manual for staging of cancer, 3rd ed. Philadelphia, Lippincott.
[2] Tarazi, R. Y., Hermann, R. E., *et al.* (1986). Results of surgical treatment of peri-ampullary tumors: a thirty-five year experience, *Surgery,* **100**, 716–723.
[3] Crist, D. W., Sitzman, J. V., *et al.* (1987). Improved hospital morbidity, mortality and survival after the Whipple procedure, *Ann Surg,* **206**, 358–365.
[4] Pellegrini, C. A., Heck, C. F., *et al.* (1989). An analysis of the reduced morbidity and mortality rates after pancreaticoduodenectomy, *Arch Surg,* **124**, 778–781.

[5] Yasuda, K., Mukai, H., *et al.* (1988). The use of endoscopic ultrasonography in the diagnosis and staging of carcinoma of the papilla of Vater, *Endoscopy,* **20**, 218–222.

[6] Tio, T. L., Tytgat, G. N. J., *et al.* (1990). Ampullopancreatic carcinoma: preoperative TNM classification with endosonography, *Radiology,* **175**, 455–461.

[7] Tio, T. L., Cheng, J., *et al.* (1991). Endosonographic TNM staging of extrahepatic bile duct cancer: comparison with pathological staging, *Gastroenterology,* **100**, 1351–1361.

[8] Tio, T. L., Reeders, J. W. A., *et al.* (1993). Endosonography in the clinical staging of Klatskin tumor, *Endoscopy,* **25**, 81–85.

